# Multifactorial approach is needed to unravel the maturation phases of human neurons derived from induced pluripotent stem cells

**DOI:** 10.1038/s41598-024-81140-4

**Published:** 2025-01-21

**Authors:** Maissa Ben Mahmoud, Anikó Rátkai, Krisztina Bauer, Norbert Bencsik, Attila Szücs, Katalin Schlett, Krisztián Tárnok

**Affiliations:** 1 Department of Physiology and Neurobiology, Institute of Biology, Eötvös Loránd University, Pázmány Péter Sétány 1/C, Budapest, 1117 Hungary; 2 Hungarian Centre of Excellence for Molecular Medicine, Szeged, Hungary

**Keywords:** Induced pluripotent stem cell, Human neuron, Differentiation, Maturation, Electrophysiology, Morphometry, Neural stem cells, Cellular neuroscience, Biological models, Differentiation

## Abstract

Neurons derived from induced pluripotent stem cells (h-iPSC-Ns) provide an invaluable model for studying the physiological aspects of human neuronal development under healthy and pathological conditions. However, multiple studies have demonstrated that h-iPSC-Ns exhibit a high degree of functional and epigenetic diversity. Due to the imprecise characterization and significant variation among the currently available maturation protocols, it is essential to establish a set of criteria to standardize models and accurately characterize and define the developmental properties of human neurons derived from iPSCs. In this study, we conducted comprehensive cellular and network level analysis of the functional development of human neurons, generated from iPSCs obtained from healthy young female peripheral blood mononuclear cells by BDNF and GDNF treatment. We provide a thorough description of the maturation process of h-iPSC-Ns over a 10-week in vitro period using conventional whole-cell patch clamp and dynamic clamp techniques, alongside with morphometry and immunocytochemistry. Additionally, we utilized calcium imaging to monitor the progression of synaptic activity and network communication. At the single cell level, human neurons exhibited gradually decreasing membrane resistance in parallel with improved excitability. By the fifth week of maturation, firing profiles were consistent with those of mature regular firing type of neurons. At the network level, fast glutamatergic and depolarizing GABAergic synaptic connections were abundant together with synchronized network activity from the sixth week of maturation. Alterations in the expression of GABA_A_ receptor subunits were also observed during the process of maturation. The sequence of differentiation events was consistent, providing a robust temporal framework to execute experiments at defined stages of neuronal maturation as well as to use a specific set of experiments to assess a culture’s maturation. The uncovered progression of differentiation events provides a powerful tool to aid the planning and designing of targeted experiments during defined stages of neuronal maturation.

## Introduction

Induced pluripotent stem cell (iPSC) technology, developed by Yamanaka and Takahashi in 2006, provides a valuable tool to circumvent the ethical and technical difficulties involved in using living human brain tissue. It also offers an opportunity to overcome the inadequacies of animal models used in neurodegenerative disease studies, which only partially recapitulate the precise mechanisms of disease development and progression, especially in late-onset diseases^1^. iPSCs are generated in vitro from somatic cells, mostly from skin or blood, by ‘reprogramming’ them to an embryonic pluripotent state. This broad developmental potential allows the generation of human cells, such as neurons, for therapeutic purposes or the construction of disease models, enabling the analysis of disease aspects that cannot be studied in patients or animal models^2^. Such stem cell-based test systems have several key advantages over traditional animal models, including the ability to (i) study and characterize human-specific, disease-related cell types that model early aspects of human brain development^3^, (ii) reflect the genetic background of patient groups^4^, and (iii) be used for high-throughput screening of drug candidates^5^.

Neurons derived from human iPSCs (h-iPSC-Ns) have been proven particularly useful in elucidating the complex molecular mechanisms of neurodevelopmental disorders and monogenic brain diseases^6–8^. Several studies have investigated the electrophysiological and morphological properties of h-iPSC-Ns^9–11^. It has been demonstrated that h-iPSC-Ns exhibit electrical properties comparable to those observed in mature neurons, including action potentials, synaptic currents and spontaneous firing, as well as morphological maturation of cultured neurons^7^. However, studies have also shown that iPSC derived neurons are functionally and epigenetically diverse^12^. Multiple differentiation and maturation protocols of h-iPSC-Ns derived from different somatic cell types have resulted in only approximate time points^10^ when neurons are regarded as ‘mature’. This is based on a successive progression of selected neuronal hallmarks (morphological, electrophysiological or gene expression characteristics) which seem to reproduce a given set of neuronal functions observed in the developing neuronal network during the early and middle stages of neuronal development. On the other hand, immature characteristics pose a challenge when using h-iPSC-Ns to study age-related pathophenotypes^13,14^. Due to the imprecise categorization and significant disparity among existing maturation protocols, standardization of the models is necessary. Therefore, it is crucial to define and establish a set of criteria for characterizing the developmental properties of neurons derived from iPSCs^15^.

In the present study, we have conducted a detailed characterization of h-iPSC-Ns generated from peripheral mononuclear blood cells (PMBCs) of young adult, heathy, neurotypical female volunteer by combining individual cell- and network-level experiments allowing a profound description of the in vitro maturation process of induced neurons over 10 weeks. Specifically, we have utilized conventional whole-cell patch clamp and dynamic clamp to evaluate the individual properties of the maturing h-iPSC-Ns, and calcium imaging to follow the development of the synaptic activity and network communication. Additionally, through immunocytochemistry we characterized the morphological properties of the derived neurons. All these aspects can be used as a follow-up standard of the h-iPSC-Ns maturation.

## Materials and methods

### Cell culture

Human Neural Presursor Cells (NPCs) from a healthy neurotypical young adult female volunteer were independently generated and kindly provided by the Gedeon Richter Plc., Hungary. Briefly, peripheral blood was collected from the volunteer, and peripheral mononuclear blood cells (PBMCs) were isolated using the Vacutainer^®^ CPT^™^ Cell Preparation Tube with Sodium Heparin (BD Biosciences). After in vitro expansion, PBMCs were transduced using the CytoTune^®^-iPS 2.0 Sendai Reprogramming Kit (Thermo Fisher Scientific), following the manufacturer’s instructions. The colonies with ES-like appearance were manually isolated between Day 21 to Day 27 post-transduction and cultured as iPSCs thereafter^16,17^. NPCs were generated from the iPSC cells using dual SMAD inhibition protocol (published previously by Nagy et al., referred to the clone as CTL1 S11) which give rise to forebrain cortical glutamatergic neurons under GDNF and BDNF induction^17^. iPSCs- and NPCs generation was a part of a framework study which was approved by the Research Ethics Committee of Heim Pál Childrens’ Hospital (permission number KUT-83/2013)^17^. Written informed consent had been obtained before the subject entered the original study. All research was performed in accordance with relevant guidelines/regulations.

NPCs were plated in Poly-L-Ornithine (PLO) (0.01%, Sigma, #P4957) and laminin (3 μg/cm^2^, Sigma, #L2020) coated 6-well dishes (Nunclon^™^, ThermoFisher Scientific, #140685). The culturing medium contained DMEM/F12 GlutaMAX (Gibco, #31331-028) and Neurobasal (Gibco, #21103-049) with 1% of N2 Supplement (Gibco, #17502-048), 2% of B27 Supplement (Gibco, #17504-044)), 2 mM of Glutamax (Gibco, #35050061), non-essential amino acid solution (Sigma, #M7145) and antibiotic antimycotic solution (Sigma, #A5955). 10 ng/mL fibroblast growth factor basic (FGF-basic, AA 1-155, Recombinant Human Protein #PHG0264), 10 ng/mL epithelial growth factor (EGF, Recombinant Human Protein #PHG0311) and 10 μM ROCK Inhibitor Y-27632 (Sigma, #Y0503) were added to the media freshly before use. The cells were grown to confluence, then passaged after six or seven days. For terminal differentiation, 10^5^ cells / well were plated into 24-well plates initially containing glass coverslips (12 mm diameter, Paul Marienfeld GmbH & Co.KG, #0111520) coated with PLO for 1 h at 37 °C. After 2 times wash with PBS, 12 μg/mL of laminin (500 μL, Sigma, L2020) was added to the wells for overnight at 37 °C, which was replaced by culturing medium before the cell seeding. After 24 h, culturing medium was changed to the differentiation medium which contained BrainPhys Neuronal Medium (StemCell Technologies, #05790) with 1% of N2 Supplement, 2% of B27 Supplement and 1 μg/mL laminin, 10 ng/mL brain derived neurotrophic factors (BDNF, Gibco, #PHC7074), 10 ng/mL glial derived neurotrophic factor (GDNF, Gibco, #PHC7045), 1 mM dibutyryl cyclic-AMP (Sigma, #D0627) and 200 nM ascorbic acid (Sigma, #A8960) were added freshly to the medium and a half medium change was performed twice a week during the differentiation.

### Immunocytochemistry

h-iPSC-Ns were fixed between the 1st and 7th week (DIV8 to DIV51) with 4% PFA in phosphate-based saline (PBS, pH 7.4) for 20 min and permeabilized with 0.1% Triton-X100 for 5 min at room temperature. Blocking was performed for 1 h in 2% BSA at room temperature, followed by an overnight incubation with primary antibodies in 2% BSA at 4 °C. After thorough wash with PBS, cultures were incubated for 1 h with secondary antibodies at room temperature (see Table 1 for details). Cells were mounted in Mowiol 4–88 (Polysciences) with DAPI. Images were acquired with a Zeiss CellObserver Z1 widefield fluorescent microscope, using Plan-Apochromat 20×/0.8, LD Plan-Neofluar 20×/0.4 and Plan-Apochromat 63×/1.4 oil (Zeiss) objectives. Images were acquired as z-stack tiles with Axiocam MRm camera (Zeiss) using the ZEN software (version 2.6, Zeiss).

**Table 1:** List and characterization of the used antibodies.

Primary antibodies (source)	Provider, cat number	Dilution
Anti-GFAP (rabbit)	Synaptic system, #173 002	1:1000
Anti-βIII-tubulin (mouse)	Exbio, #11-427-C100	1:1000
Anti-ankyrin-G (mouse)	Santa Cruz biotechnology, #sc-12719	1:300
Anti-MAP2 (rabbit)	Synaptic system, #188003	1:1000
Anti-Synapsin1 (mouse)	Sigma, #MABN1847	1:1000
Anti-Shank2 (guinea-pig)	Synaptic system, #162204	1:500
**Secondary antibodies**		
Goat anti-rabbit alexa fluor 488	Invitrogen, #11008	1:500
Goat anti-mouse alexa Fluor 488	Invitrogen, #A10684	1:500
Goat anti-mouse alexa fluor 647	Invitrogen, #A21237	1:500
Goat anti-guinea-pig alexa fluor 633	Invitrogen, #A21105	1:500
Streptavidin-TRITC	Jackson immunoresearch, #016-020-084	1:300

### Sholl analysis

Neuronal shape was traced by the Simple Neurite Tracer plugin^18^ with Fiji (ImajeJ framework) software^19^ (https://imagej.net/plugins/snt/) on the microscopy images collected from three independent neuronal induction, where cells were filled up with biocytin. A line stack of the traces was created for subsequent Sholl analysis, which was performed using the Sholl analysis plugin (https://imagej.net/plugins/sholl-analysis) with a starting radius of 10 μm and increments of 10 μm until the farthest point of the dendrites. Data relating to neurite length and intersection were obtained using the Simple Neurite Tracer plugin.

### Patch clamp recording and analyses

Electrophysiological recordings were performed under an Axiovert 200 M microscope (Zeiss) equipped with an EC Plan-Neofluar 20×/0.5 objective. Spontaneous synaptic activity and evoked responses were recorded at room temperature (21–23 °C) in whole-cell conditions using a MultiClamp 700B amplifier (Molecular Devices). Intracellular voltage and current traces were sampled at 20 kHz and stimulus command waveforms were generated by the data acquisition software DASYLab v.11 (National Instruments). Patch pipettes (7–10 MOhm) were pulled from standard wall glass of 1.5 mm OD (World Precision Instruments). The composition of the bath solution (ACSF) was (in mM): NaCl 140, KCl 5, CaCl_2_ 2, MgCl_2_ 1, HEPES 5, D-glucose 10; pH set to 7.45, while patch electrodes were filled with the following solution (in mM): K-gluconate 100, KCl 10, KOH 20, MgCl_2_ 2, NaCl 2, HEPES 10, EGTA 0.2, D-glucose 5; pH set to 7.3. For subsequent morphological analysis, 0.5 mM biocytin (Tocris, #33-491-0) was added to the patch pipette solution. A current step stimulation protocol was used to record voltage responses by applying stepwise current commands of 350 ms duration, starting at – 20 pA and incremented by + 2 pA were delivered in 1.25 s cycles. Multiple physiological parameters including the input resistance, membrane time constant, spike amplitude and half-width were determined for each recorded cell obtained from 2 to 5 independent neuronal inductions.

Spontaneous excitatory postsynaptic currents (sEPSCs) were acquired at − 60 mV holding potential in voltage clamp mode. To characterize the chemical properties of the postsynaptic currents, selective pharmacological blocking of AMPA, NMDA and GABA_A_ receptors was carried out by using CNQX (10 μM, Tocris, #1045), AP-5 (40 μM, Tocris, #3693) and bicuculline (30 μM, Tocris, #0131), respectively. Analysis of both the sEPSCs and the evoked responses was performed using NeuroExpress software developed by A. Szücs.

(https://www.researchgate.net/publication/323547374_Analysis_of_miniature_excitatory_postsynaptic_currents_mini_analysis_in_NeuroExpress_Program_available_for_download).

### Dynamic clamp experiments

We used the dynamic clamp technique to investigate the neurons’ firing responses under physiologically more realistic inputs by exposing the recorded neurons to simulated oscillatory synaptic inputs consisting of one excitatory and one inhibitory channel^20^. The frequency of the oscillatory envelope of the excitatory synaptic input was either 4 Hz (*low frequency* stimulation) or 20 Hz (*high frequency* stimulation). Stimulus waveforms serving as presynaptic voltage for the dynamic clamp system were generated by the data acquisition software DASYLab v.11 (National Instruments). The StdpC v.2012 program was used to compute the synaptic conductance and the net postsynaptic current^21^. Synaptic time constant was 10 ms for both the excitatory and inhibitory connections, while their reversal potential was 0 mV and − 72 mV, respectively. The excitatory synaptic conductance (matching that of the value of the inhibitory conductance) was set in a way that approximately 10 action potentials were evoked under a single sweep of the oscillatory theta waveform. The data were collected from three to four independent neuronal inductions in weeks 4, 6, 8 and 10.

### Calcium imaging

h-iPSC-Ns cultivated on glass cover coverslips at 4, 6 and 8 weeks of maturation were loaded with 2.5 µM Fluo-3-AM (Invitrogen, #F1242) dissolved in differentiation medium for 30 min at 37 °C and at 5% CO_2_. For the image acquisition, BrainPhys^™^ Imaging Optimized Medium (StemCell Technologies, #05796) was used in an environmental chamber at 37 °C and with 5% CO_2_. Fluo-3-AM was excited with a combination of 488 and 514 nm excitation laser wavelengths with a 520–560 nm emission filter. Images were captured for 5 min with a 500 ms frame rate using a Zeiss Axio Observer SD spinning disc confocal microscope equipped with a LCI Plan-Apochromat 25×/0.8 oil objective. Data were analyzed using the Mesmerize software^22^. Somatic regions of interest (ROIs) were defined over every cell within the field of view. Mean fluorescent intensities of the ROIs were expressed as ΔF/F0 ratio, which represents the change in fluorescent intensity (ΔF) normalized to the baseline fluorescence of Fluo-3 (F0). Based on the calcium transients (ΔF/F0), heatmaps were generated, where results are depicted using a standard z score (0–6) and a color scale, where 0 denotes the lack of a Ca^2+^ transient and 6 represents the maximum relative intensity of calcium transients.

To detect synchronization of Ca^2+^ transients as indicatives of the development of robust synaptic connectivity, we calculated pairwise Pearson correlations for the ROI channels. First we established the sequences of the arrival times of Ca^2+^ waves and created event density functions by convolving these arrival times with a Gaussian function (kernel, 5 s half-width). The correlation coefficients for such smooth and continuous density functions were calculated and plotted in the form of the correlation matrix. Averaged values obtained from independent experiments were used to calculate the interevent interval, peak halfwidth and Spearman correlation of the mentioned independent variables.

### Quantitative real-time PCR (qRT-PCR)

Cultures were lysed and RNA samples were obtained using Quick‐RNA MiniPrep (ZYMO Research, USA). Total RNA was quantified with a spectrophotometer (Implen, NanoPhotometer N60). Reverse transcription was performed with the Maxima First Strand cDNA Synthesis for RT-qPCR kit (Thermo Scientific, #K1672) according to the manufacturer’s instruction. Messenger RNA expression was investigated using the Maxima SYBR qPCR Master Mix (Thermo Scientific, #K0252) with specific primers (Table 2). qPCR was performed using a CFX96 (C1000 Touch) from Bio‐Rad Laboratories (USA) with the following settings: 1 cycle at 95 °C for 10 min, 40 cycles at 95 °C for 15 s followed by 55 °C for 30 s and 72 °C for 30 s. Cq and RFU values were obtained from Bio‐Rad CFX Manager software (Bio-Rad, USA). Cq values were normalized to housekeeping genes GAPDH and RLP-13a. The relative expression of the given genes was calculated using the $\Delta \Delta {\text{Cq}}$ method from triplicated samples using the CFX Manager software (version 3.1, BioRad).

**Table 2:** Primer sequences used in RT-qPCR experiments.

Gene name, ID	Forward sequence (5′–3′)	Reverse sequence (3′–5′)
GABARα1: NM_001127645.2.1	GGATTGGGAGAGCGTGTAACC	TGAAACGGGTCCGAAACTG
GABARα2: NM_000807.4	GTTCAAGCTGAATGCCCAAT	ACCTAGAGCCATCAGGAGCA
GABARα5: NM_000810.4	CTTCTCGGCGCTGATAGAGT	CGCTTTTTCTTGATCTTGGC
GABARβ2: NM_001371727.1	GCAGAGTGTCAATGACCCTAGT	GCAGAGTGTCAATGACCCTAGT
GABARβ3 : NM_000814.6	CCGTTCAAAGAGCGAAAGCAACCG	TCGCCAATGCCGCCTGAGAC
GABARγ2 : NM_198904.4	TCGCCAATGCCGCCTGAGAC	GGCAGGAGTGTTCATCCATT
GABARγ3: NM_033223.5	AACCAACCACCACGAAGAAGA	CCTCATGTCCAGGAGGGAAT
SLC12A2: NM_001046.3	CAGCCCTCCAGAATGGTACT	CAACTTTCCTGTGTGCTTTCA
SLC12A5: NM_020708.5	GGTGAGACAAGGGTCCAACT	CTTGCATAAGAGCCGATGAG
GAPDH: NM_002046.7	ACCACAGTCCATGCCATCAC	TCCACCACCCTGTTGCTGTA
RLP-13a: NM_012423.4	CAAGCGGATGAACACCAAC	TGTGGGGCAGCATACCTC

### Statistical analyses of the data

One-way ANOVA, Turkey post-hoc test, or non-parametric Mann–Whitney tests were used for statistical evaluation unless otherwise indicated. SPSS Statistics (version 25, IBM, https://www.ibm.com/analytics/spss-statistics-software) was used to calculate statistics. Data are displayed as mean ± SEM, unless otherwise indicated. p values were accepted statistically significant as *p* < 0.05 (*), *p* < 0.01 (**), *p* < 0.001 (***).

## Results

### Morphological maturation of human iPSC-derived neurons is prominent after 5 weeks

To track the development and maturation of human cortical neurons in vitro, we used a formerly characterized induced pluripotent stem cell (iPSC) line (CTL1 S11) originated from a neurotypical, young adult female person. The S11 iPSC cells can be differentiated into forebrain neurons and astroglia producing neuronal precursor cells (NPCs) by the 2-inhibitor method^23^. Human neurons were differentiated from S11 NPCS in neuronal differentiation medium supplemented with BDNF (10 ng/mL), GDNF (10 ng/mL) and dibutyryl-cAMP (1 mM) and were cultured for 10 weeks, in vitro.

**Fig. 1:**
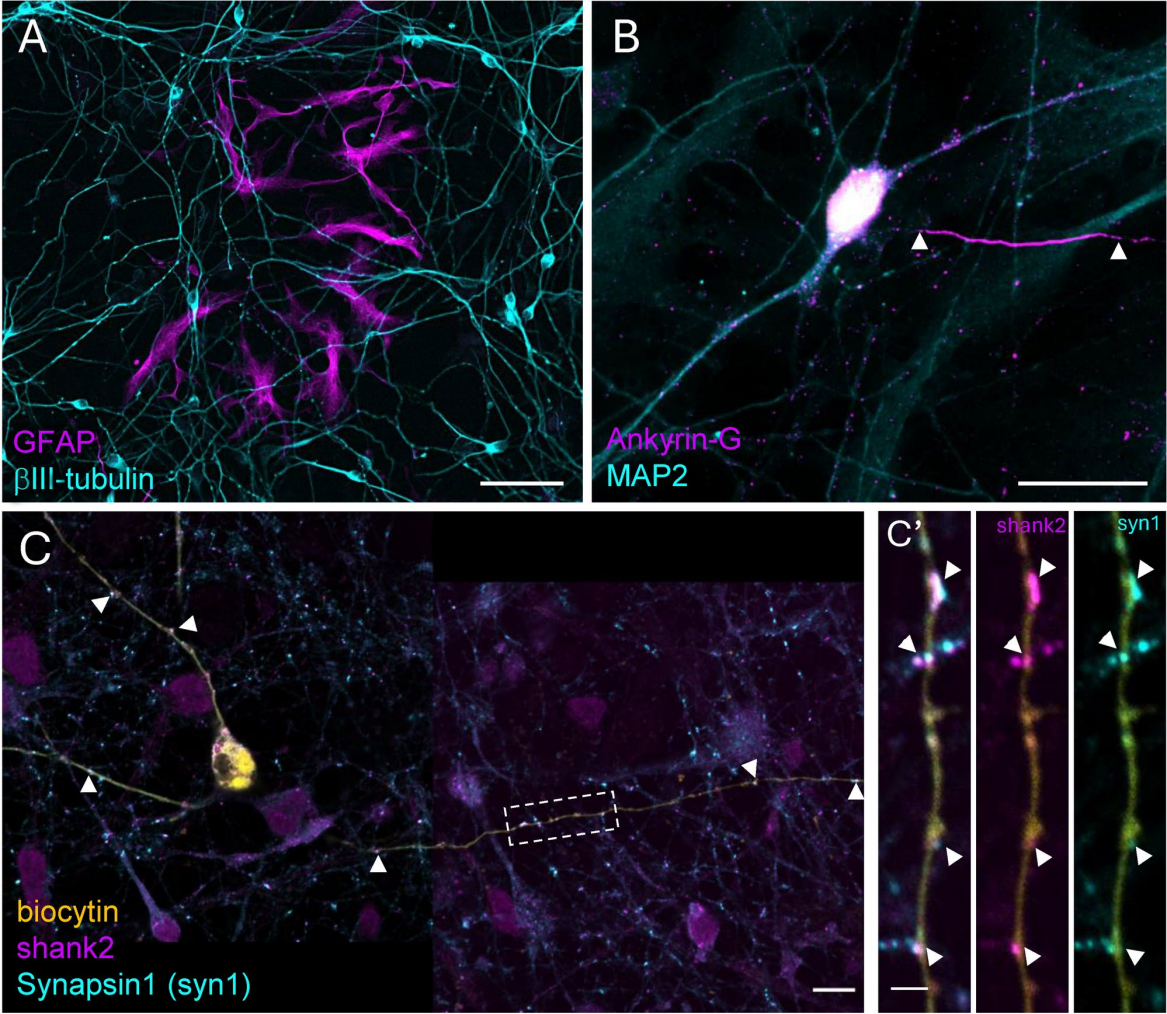
Differentiated h-iPSC-N cultures express neuronal and glia markers from the first week of maturation. (**A**) βIII-tubulin and GFAP immunopositive neurons and glia cells, respectively, were identified from the first week of the differentiation. (**B**) Ankyrin-G positivity clearly indicated the axon initial segment (AIS) at 3 weeks of differentiation, while MAP2 staining highlighted the dendrites. Arrowheads designate the length of the AIS. (**C**) Biocytin filled up cells showed immunopositivity for the postsynaptic marker Shank2 at 4 weeks of differentiation. Close appositions with the presynaptic marker Synapsin1 are indicated by arrowheads. (**C’**) Enlarged segment from (**C**) (see the dashed area); arrows depict individual postsynaptic Shank2 puncta (shank2) in the close vicinity of presynaptic Synapsin1 (syn1) labelling. Scale bars represent 50 μm (**A**), 20 μm (**B**), 10 μm (**C**) or 2 μm (**C’**).

**Fig. 2:**
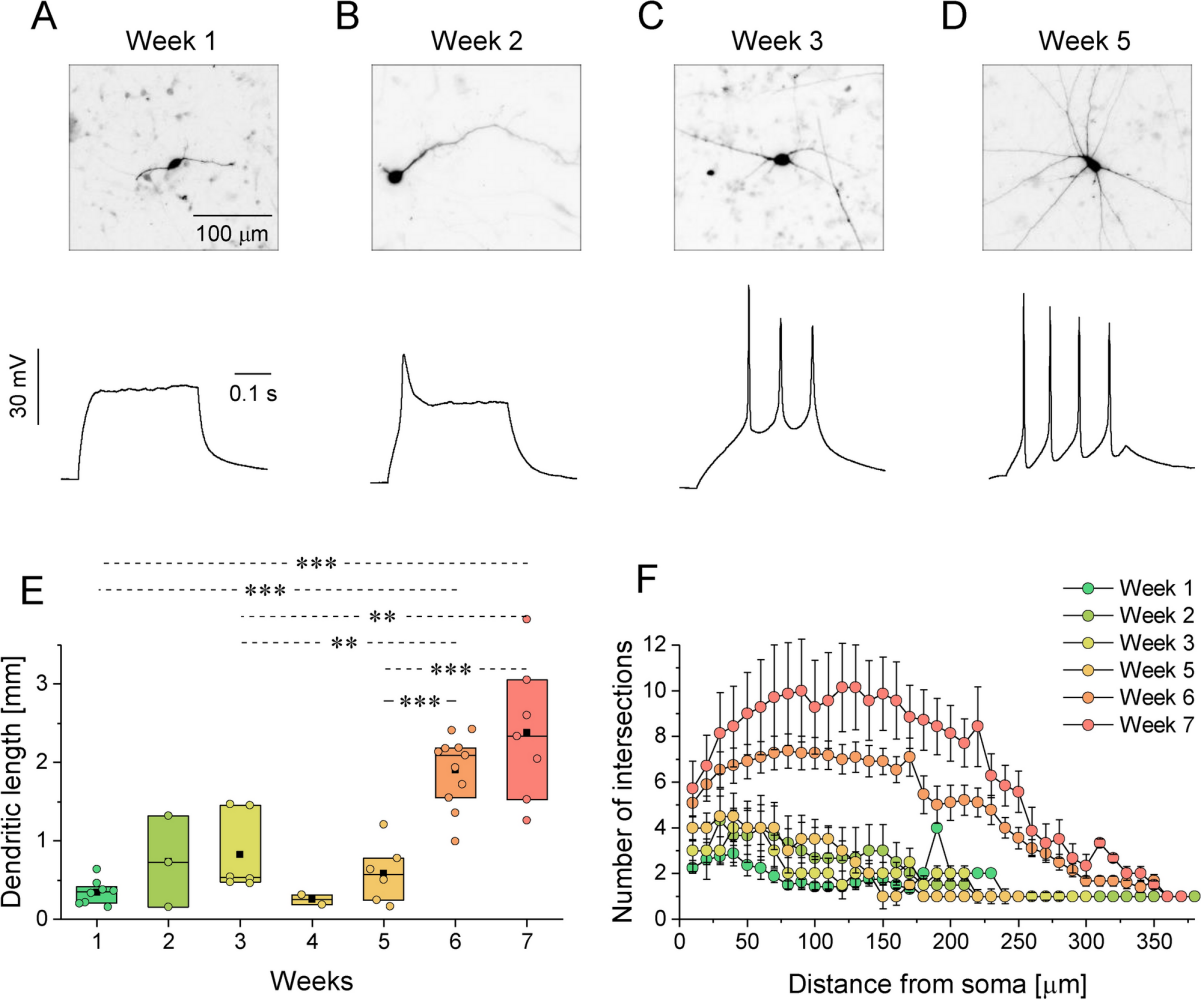
Electrophysiological and morphological properties develop in a time-dependent manner. (**A**–**D**) Cells filled with biocytin during patch clamp recordings were used for the morphological study. Voltage responses were recorded using standard current clamp protocols under whole-cell condition. The arborization of the biocytin + cells was characterized by Sholl analysis, displaying (**E**) the total dendritic length and (**F**) the arborization of neural processes represented as the number of intersections in the function of the distance from the soma. Data are presented as box plots of interquartile range together with individual data points (from 3 independent neuronal inductions; n = 8, 3, 6, 2, 6, 11, 7 for week 1–7, respectively). Represented by Turkey post-hoc test, *p* < 0.05(*), *p* < 0.01(**), *p* < 0.001(***). Normalized branching data (intersections per a given radius) are presented as mean ± SE.

**Fig. 3:**
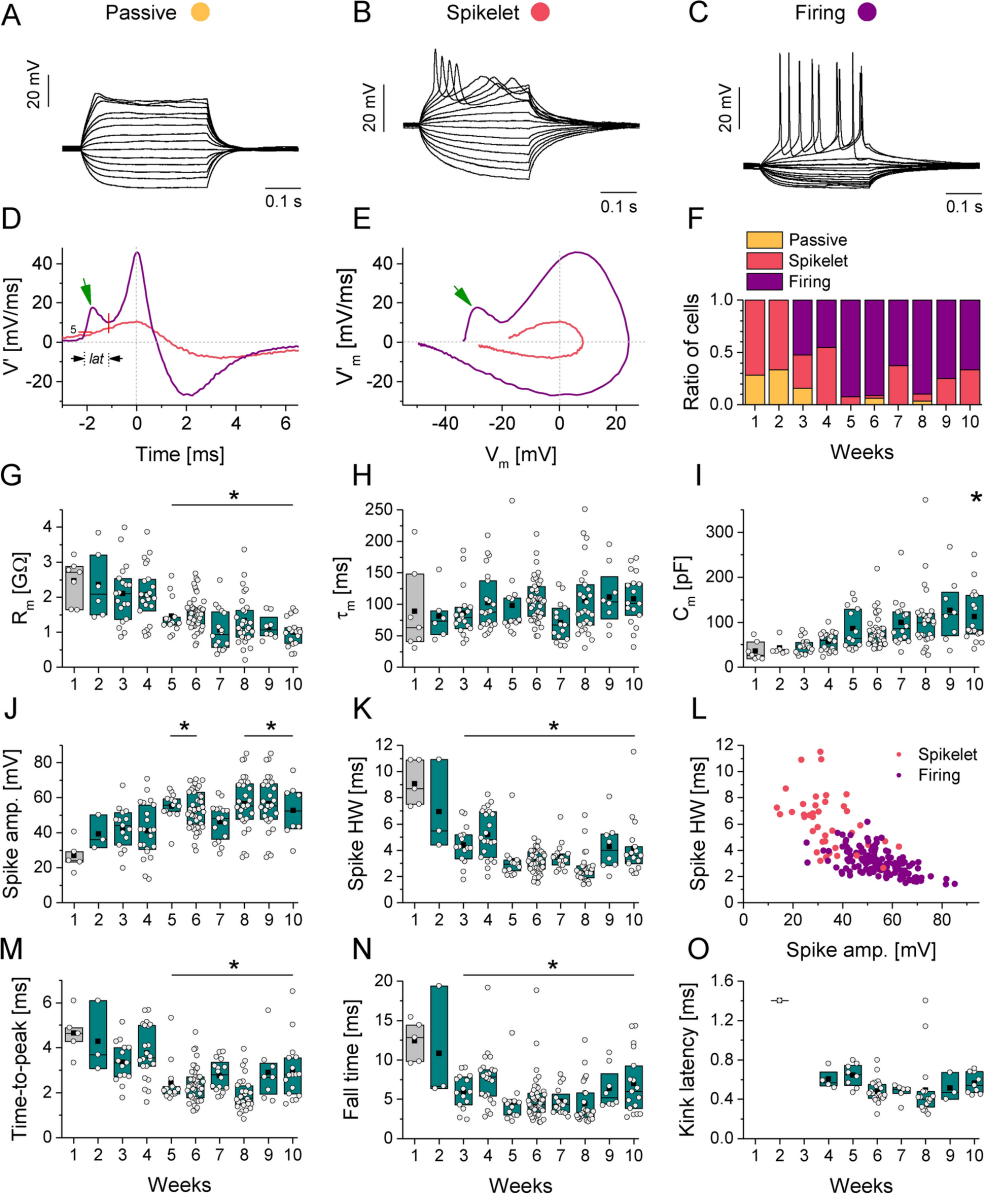
Evolution of passive and active membrane properties during h-iPSC-Ns maturation. (**A**–**C**) Three distinct electrophysiological phenotypes—passive (**A**), single spiking/spikelet (**B**) and firing (**C**)—were observed under current clamp protocols in whole cell condition during maturation. (**D**) First derivative of the membrane potential and the phase portrait (**E**) differ between single spiking/spikelet (red) vs. firing (purple) phenotypes. The green arrows indicate the prominent transient fluctuation of the action potential slope (*kink*) in the firing phenotype. **F** Ratios of the three electrophysiological phenotypes over time during 10 weeks of maturation. (**G**–**I**) Passive membrane properties during 10 weeks of maturation. (**G**) Average membrane resistance (R_m_) decreased over time. (**H**) and (**I**) Membrane time constant (τ_m_) and the membrane capacitance (C_m_) increased during the 10 weeks of maturation. (**J**), (**K**), (**M**), (**N**) and (**O**) Active membrane properties during 10 weeks of maturation. (**J**) Spike amplitude of action potentials showed a time dependent increase with a stabilization phase around the 5 weeks of maturation. Spike half-width (**K**), time-to-peak (**M**) and fall time (**N**) decreased in a time-dependent manner. (**L**) Negative correlation between the spike amplitude and half-width of the firing phenotypes is observed, separating the spikelet and firing types. (**O**) Kinks can be detected, where kink latency values can be calculated for the firing type h-iPSC-Ns from the 4th week of maturation. Data presented as box plots of interquartile range together with individual data points (obtained from 2 to 5 independent neuronal inductions; n = 7, 6, 19, 22, 13, 46, 16, 29, 8, 18 for week 1–10, respectively). Significancy is represented by One-way ANOVA and Tukey post-hoc test, *p* < 0.05 (*). All the datasets were compared to the first week values representing the baseline for the statistical analysis with the corresponding boxplot colored in gray.

The presence of neuron-specific markers, βIII-tubulin and MAP2 were detected from the first week in culture (Fig. 1A, B, Supplementary Fig. S1). By this time, glial fibrillary acidic protein (GFAP) positive glia cells were also present (Fig. 1A) in small groups. The density of neurites gradually increased until the 3rd week of differentiation, while the dispersion of the glia cells got more homogenous. From the 4th week on, the basal glia layer was confluent, supporting a dense neuronal network (see Supplementary Fig. S1). The formation of the axon initial segment (AIS) in the vicinity of the soma was detected by ankyrin-G staining from the 3rd week of cultivation (Fig. 1B). Putative synaptic connections were identified at the 4th week of cultivation by detecting respective pre- and postsynaptic markers Synapsin1 and Shank2 in close proximity (Fig. 1C,C′). Developing cultures were subject to whole-cell patch clamp measurements between week 1 and 10 (Figs. 2, 3). Some of the measured cells were filled with biocytin (Fig. 1C), enabling the morphometric characterization of the developing neurons in parallel with determining their electrophysiological profiles (see the recorded traces below the corresponding images on Fig. 2A–D). Based on the visualization of the biocytin signal, we reconstructed and analyzed the cell morphology within a 375 μm radius from the soma (Fig. 2). Sholl analyses indicate that during the maturation of h-iPSC-Ns, both the total dendritic length and the complexity of arborization increase in a biphasic manner (Fig. 2E,F). The initial process expansion was slowly followed by the branching of processes, however after the 5th week of cultivation, elongation and branching accelerated, leading to more elaborate morphology (Fig. 2D) together with the appearance of more mature electrophysiological properties in the neurons (Fig. 3).

### Multifactorial analysis reveals the timescale of stabilizing neuronal electrophysiological properties during maturation

Patch clamp measurements in whole-cell configuration were performed weekly from the 1st week (DIV8, days in vitro) to the 10th week (DIV74) to determine the intrinsic biophysical properties and the excitability of h-iPSC-Ns under incrementing levels of injected current (Fig. 3A–C). The physiological parameters and intrinsic excitability of h-iPSC-Ns were analyzed as functions of the maturation time (Fig. 3G–O). Initially, most h-iPSC-Ns exhibited passive (resistive-capacitive-type) membrane response (Fig. 3A,F) indicating the lack of voltage-dependent membrane currents. The average membrane resistance was at maximal level in the first week of maturation and gradually decreased over time (Fig. 3G). The membrane time constant (τ_m_) remained consistent across our experiments (Fig. 3H), however, we observed a monotonous increase of the calculated membrane capacitance during the 10 weeks (Fig. 3I). This observation agrees with the morphological maturation of the cells, i.e. the development of extended processes and increase of total membrane surface. From the 3rd week, h-iPSC-Ns displayed action potentials first with single spike/spikelet responses (Fig. 3B, named as “spikelets”) and later with repetitive firing (Fig. 3C, designated as “firing”) under depolarizing current steps.

The percentage of firing type neurons increased over time (Fig. 3F). Action potentials became gradually more pronounced, and sharper as revealed by the analysis of the first-time derivative of the membrane potential (Fig. 3D,E). Single spiking/spikelet neurons typically emitted action potentials with slow dynamics (slope remaining below 20 mV/ms; see the red curves). The phase portrait on Fig. 3E also demonstrates the higher amplitude and faster dynamics of the action potentials in the case of the firing phenotype (see the purple curves). We also noticed the high abundance of firing responses with a characteristic bump (also referred to as *kink*) in the phase portraits of action potentials (Fig. 3D,E green arrow). This feature indicates a temporal mismatch between the activation of transient Na^+^-currents in the soma vs. axon initial segment^24^. By detecting the onset and termination of the kink in the action potential waveforms (Fig. 3D, *lat*) we extracted a new parameter called kink latency and found that it was stable across our measurements after 4 weeks in vitro (Fig. 3O). Our patch clamp measurements also revealed a time-dependent increase of the spike amplitude (Fig. 3J) and decrease in the half-width of action potentials (Fig. 3K), in agreement with the more mature firing properties. Here, steep changes were observed until approximately 5 weeks of maturation and then parameters stabilized with less variability. Spike rise times (time-to-peak, Fig. 3M) and spike fall times (Fig. 3N) exhibited a similar biphasic change over the time of the study.

Interestingly, by examining the correlation between spike amplitude and half-width, we found a clear separation of single spiking/spikelet vs. firing neurons, as members of the two phenotypes formed distinct clusters (Fig. 3L).

### iPSC-Ns fire reliably under simulated synaptic inputs from the fourth week of maturation

**Fig. 4:**
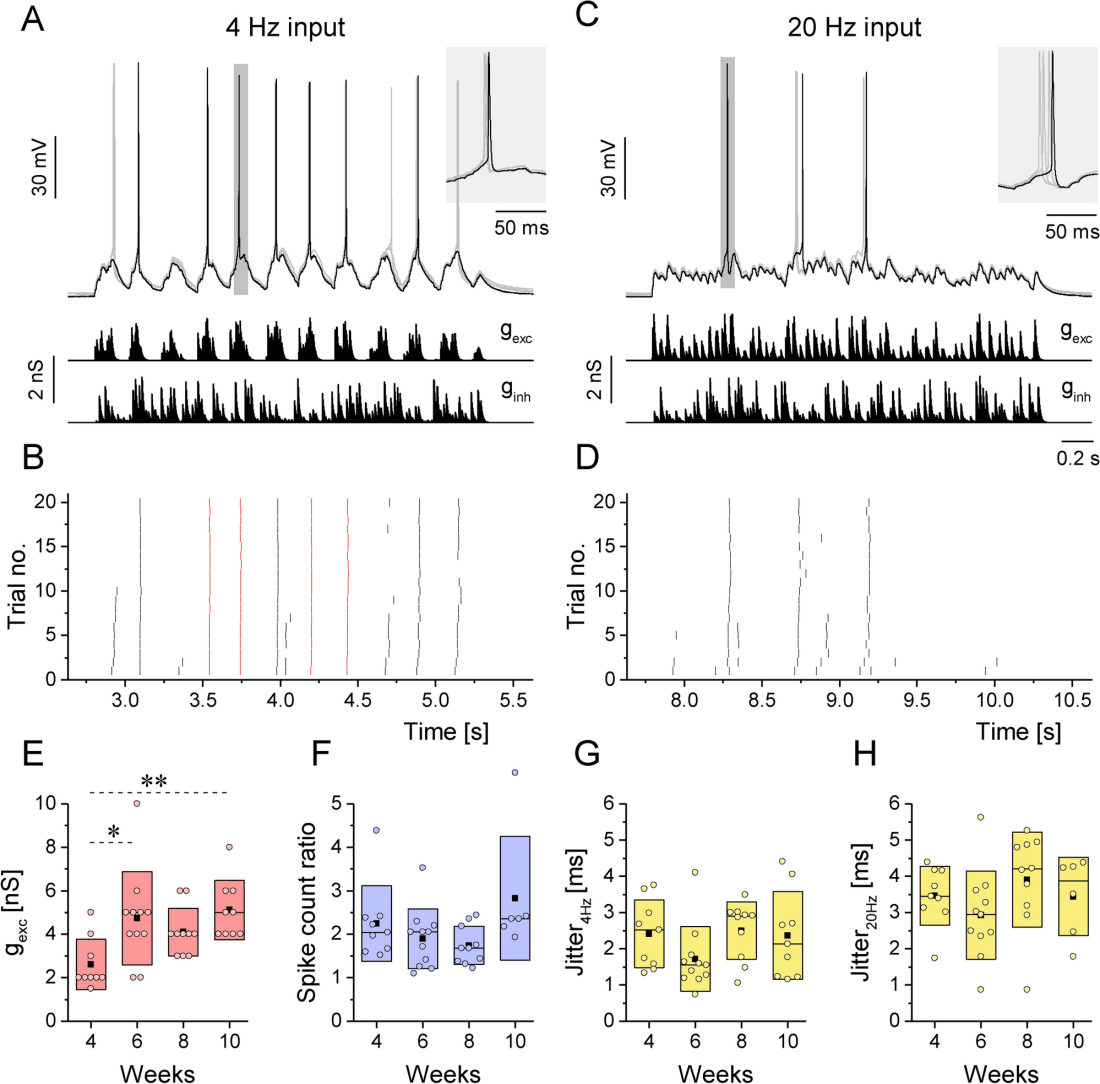
Effects of simulated oscillatory synaptic inputs on h-iPSC-Ns under dynamic clamp condition. (**A**) Four overlapping voltage traces of the neurons under simulated 4 Hz oscillation. Conductance waveforms of the excitatory (g_exc_) and the inhibitory (g_inh_) inputs are shown below the traces. (**B**) Peri-stimulus spike raster plot of the firing response of the neurons under 4 Hz oscillation. Red spikes indicate events with spike jitter less than 2 ms. (**C**) Four overlapping voltage traces of the neurons under simulated 20 Hz inputs. (**D**) Corresponding peri-stimulus spike raster plot for the 20 Hz oscillation. (**E**) Excitatory synaptic conductance required for the emission of approximately 10 spikes under the 4 Hz inputs (inhibitory synaptic strength matches that of the excitation). More mature h-iPSC-Ns required stronger excitatory or inhibitory conductance to fire the targeted spike number. (**F**) In average, twice as many spikes were evoked by the 4 Hz input than by the 20 Hz input (4 Hz/20 Hz spike count ratio). These ratios were stable during maturation (4, 6, 8 and 10 weeks). (**G**) and (**H**) Spike jitter upon 4 Hz (**G**) and 20 Hz (**H**) inputs indicate higher timing precision in case of lower frequency stimuli. Data are presented as box plots of interquartile range together with individual data points. g_exc_ (n = 9, 11, 10, 8), Spike count ratio (n = 9, 11, 9, 6), Jitter 4 Hz (n = 9, 11, 10, 9), Jitter 20 Hz (n = 9, 11, 10, 6) respectively for week 4, 6, 8 and 10 from 3–4 independent neuronal inductions. Pairwise comparison adjusted by Bonferroni correction for multiple tests, *p* < 0.05 (*), *p* < 0.01 (**).

To further characterize the excitability and integrative properties of h-iPSC-Ns, we exposed them to simulated synaptic inputs via dynamic clamp^25^. In this configuration, we can mimic in vivo conditions (temporally complex synaptic inputs with amplitude fluctuations). To achieve this, we first designed excitatory and inhibitory conductance waveforms consisting of variable amplitude synaptic transients (Fig. 4A,C, g_exc_ and g_inh_). The excitatory input (g_exc_) was used to deliver oscillatory drive to the neurons either as a low frequency input (4 Hz) or a high frequency rhythm (20 Hz). The temporal parameters of the dynamic clamp stimulation were set in a way to simulate theta- or gamma frequency synaptic drive arriving from a population of excitatory neurons. The inhibitory input (g_inh_) was non-periodic and served as a random inhibitory background. We set the maximal synaptic conductance of the excitatory input in a way that neurons emitted approximately 10 spikes under one sweep of the theta stimulation. Next, we repeated the stimulation 20 times for both the 4 Hz and 20 Hz input and observed the spike responses. Under such scenario, neurons tend to fire their action potentials in well-defined locations along the stimulus (time windows) and these can be referred to as spike events. The reliability and temporal precision in such spike events can be then calculated.

As shown in Fig. 4A, a given neuron emitted its action potentials on the top of the excitatory waves and did it in a reliable fashion. Peri-stimulus spike raster plot is shown in Fig. 4B where ticks with red color indicate the spike events where the temporal jitter of spikes was under 2 ms. Interestingly, far less spikes and higher temporal jitter were found in case of high frequency stimuli (Fig. 4D) indicating that h-iPSC-Ns exhibit lower (4 Hz) frequency-preference in their firing output. The maximal synaptic conductance used for these experiments is shown in Fig. 4E. Reaching ten spikes per sweep required higher values of g_exc_ as the neurons matured. This finding agrees with the fact that the membrane resistance of the neurons tended to drop in the same time period (Fig. 3G). In addition, all tested neurons exhibited approximately 2 times more spikes under 4 Hz than 20 Hz inputs as shown by the low/high frequency ratio plot (Fig. 4F) regardless of the in vitro age. Spike jitter calculated from firing responses under low and high frequency inputs did not change significantly during the maturation (Fig. 4G,H).

### Fast excitatory AMPA- and slower depolarizing GABA-inputs are abundant in the developing h-iPSC-Ns

**Fig. 5:**
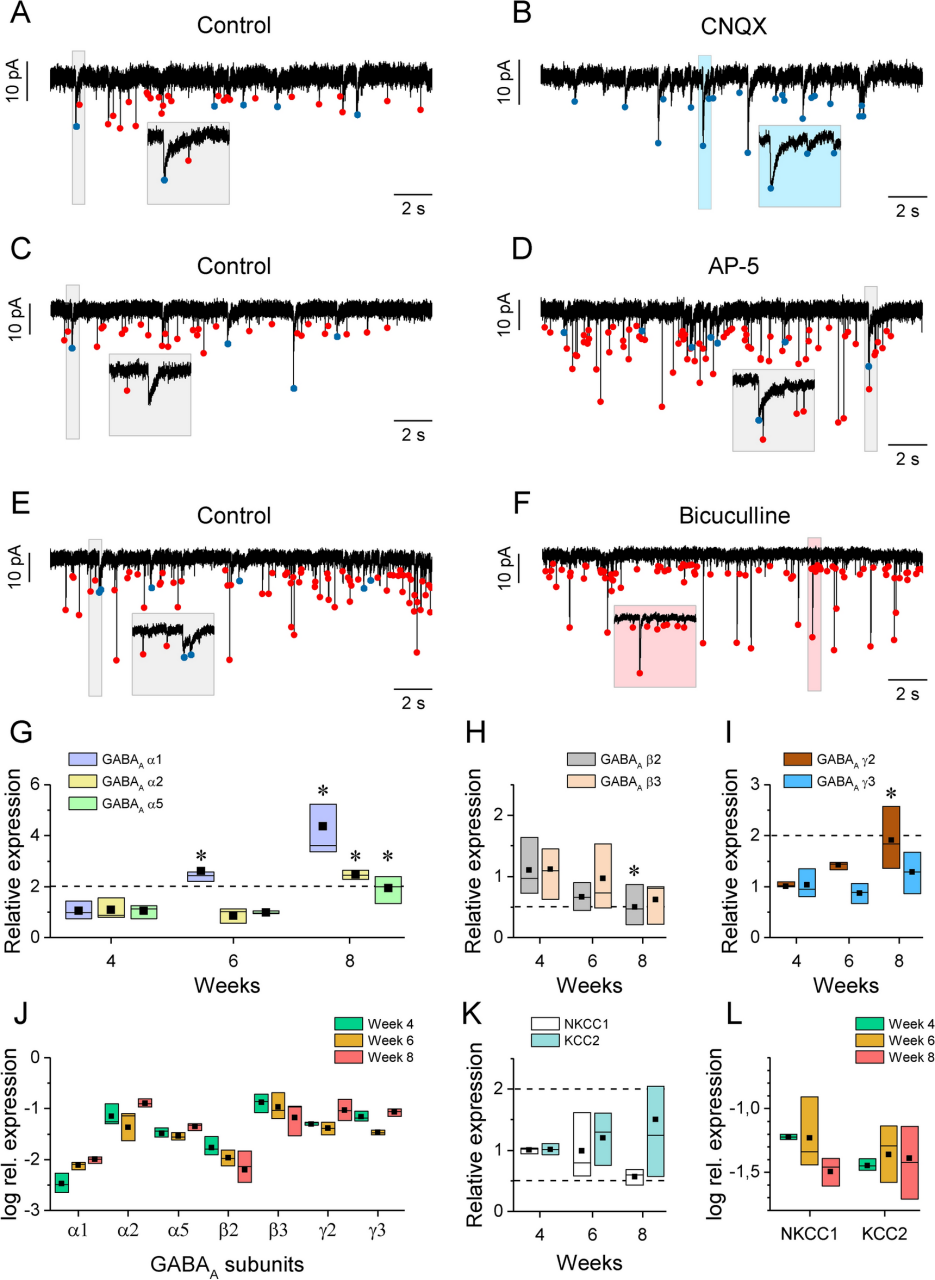
Excitatory fast glutamatergic and slow GABAergic synaptic EPSCs in h-iPSC-Ns are detected at 6th week of maturation. (**A**), (**C**) and (**E**) Slow (blue dots) and fast (red dots) spontaneous postsynaptic currents (sEPSCs) were detected in control condition. (**B**) CNQX (10 μM), a selective competitive AMPA/Kainate receptor antagonist blocked the fast sEPSCs. (**D**) Fast and slow sEPSCs persisted after application of the selective NMDA antagonist AP-5 (40 μM). (**F**) Bicuculline (30 μM), a competitive GABA_A_ receptor antagonist blocked the slow sEPSCs without affecting the fast events. GABA_A_ subunit expression changes in a time-dependent manner. (**G**) Relative expression of the GABA_A_* α*1, *α*2 and *α*5 subunits, (**H**) GABA_A_ *β*2 and *β*3 subunits and (**I**) GABA_A_ *γ*2 and *γ*3 subunits at 4, 6 and 8 weeks of maturation, normalized to gene expression level at 4 weeks. (**J**) To show comparable expression of different subunits, GABA_A_ subunit values were normalized also to the expression of human ribosomal protein L13a (*RLP13a*) at 4, 6 and 8 weeks of maturation and expressed as log rel. expression. (**K**) Relative gene expression of the *NKCC1* transporter and *KCC2* symporter at 4, 6 and 8 weeks of maturation, normalized to the 4-week values. (**L**) *NKCC1* transporter and *KCC2* symporter expression was normalized also to the logarithmic expression of the human ribosomal protein L13a at 4, 6 and 8 weeks of maturation. Data presented as box plots of interquartile range, represented by Tukey post-hoc test, *p* < 0.05 (*). The gene expression data were performed in triplicate from 3 independent cultures. Relative expression of *α* subunits (*α*1 n = 6, 6, 6; *α*2 n = 6, 4, 6; *α5* *n* = 6, 6, 6), β subunits (β2 n = 6, 6, 6; β3 n = 6, 6, 5), γ subunits (γ2 n = 6, 6, 6; γ3 n = 6, 6, 6), NKCC1 and KCC2 (NKCC1 n = 6, 6, 6; KCC2 n = 6, 6, 6), respectively, at week 4, 6, and 8 of maturation. Dashed horizontal lines indicate 0.5 × and 2 × relative expression levels.

Voltage clamp and selective blockers of putative postsynaptic receptors of glutamatergic and GABAergic neurotransmission were used to reveal the properties of synaptic communication between the developing neurons. Typically, from the 3rd week of differentiation, we observed spontaneous postsynaptic currents (sEPSCs) that exhibited either fast (decaying within 10 ms) or slower (decay > 50 ms) kinetics (Fig. 5A,C,E). CNQX (10 μM), a selective blocker of the AMPA receptors, reliably eliminated the fast kinetics events without affecting the slower ones (Fig. 5B). Using AP-5 (40 μM) to check whether NMDA-receptors contributed to the observed postsynaptic currents, did not change the appearance of the sEPSCs (Fig. 5D) while bicuculline (30 μM), a GABA_A_-receptor antagonist completely abolished the slow events, but the fast AMPA-events were not affected (Fig. 5F). We also noted that the estimated reversal potential of the slower currents (approximately − 25 mV, not shown) was more hyperpolarized than that of the AMPA-events, although all postsynaptic currents imposed a strong excitatory drive on the developing h-iPSC-Ns. Therefore, slow kinetics sEPSCs were generated by excitatory GABA transmission mimicking the early steps of neuronal development^26^ while fast sEPSCs have the characteristics of typical AMPA-type glutamatergic postsynaptic currents in mature neurons^27^.

We also investigated the changes in the gene expression levels of commonly occurring GABA_A_ ionotropic receptor subunits *(α*1, *α*2, *α*5; *β*2, *β*3; *γ*2, *γ*3) by qRT-PCR during the maturation of h-iPSC-Ns. Based on our results, all investigated subunits were expressed in h-iPSC-Ns regardless of the developmental age (Fig. 5G–I) but subunit-specific differences were found in the relative expression data during maturation. When the relative expression was normalized to data obtained from 4-week-old cultures, especially the amount of *α1* subunit showed a time-dependent change (Fig. 5G). Expression level of *β2* subunit showed a significant decrease while the *β3* had a non-significant decreasing tendency during maturation (Fig. 5H). Age-dependent increase was also observed in case of the *γ2* subunit expression (Fig. 5I) while the level of *γ3* subunit expression did not change over the study period (Fig. 5I). We also compared how the expression of the subunits varied relative to each other. In this case, the expression levels were normalized and plotted relative to that of human ribosomal protein L13a (*RLP13a*). *α2*, *β3* and *γ2/3* subunits were the most dominant in our cultures (Fig. 5J).

The depolarization inducing effect of GABA_A_ receptors depends on the function of chloride transporters^28^, therefore the expression of *NKCC1* and *KCC2* chloride transporters were also examined. While a slight but not significant decrease in the relative level of *NKCC1* expression was detected, the expression of *KCC2* was not changed during maturation, regardless of the way of normalization (Fig. 5K,L).

### Synchronized network activity is developed with a delay compared to the stabilization of individual electrophysiological phenotypes

**Fig. 6:**
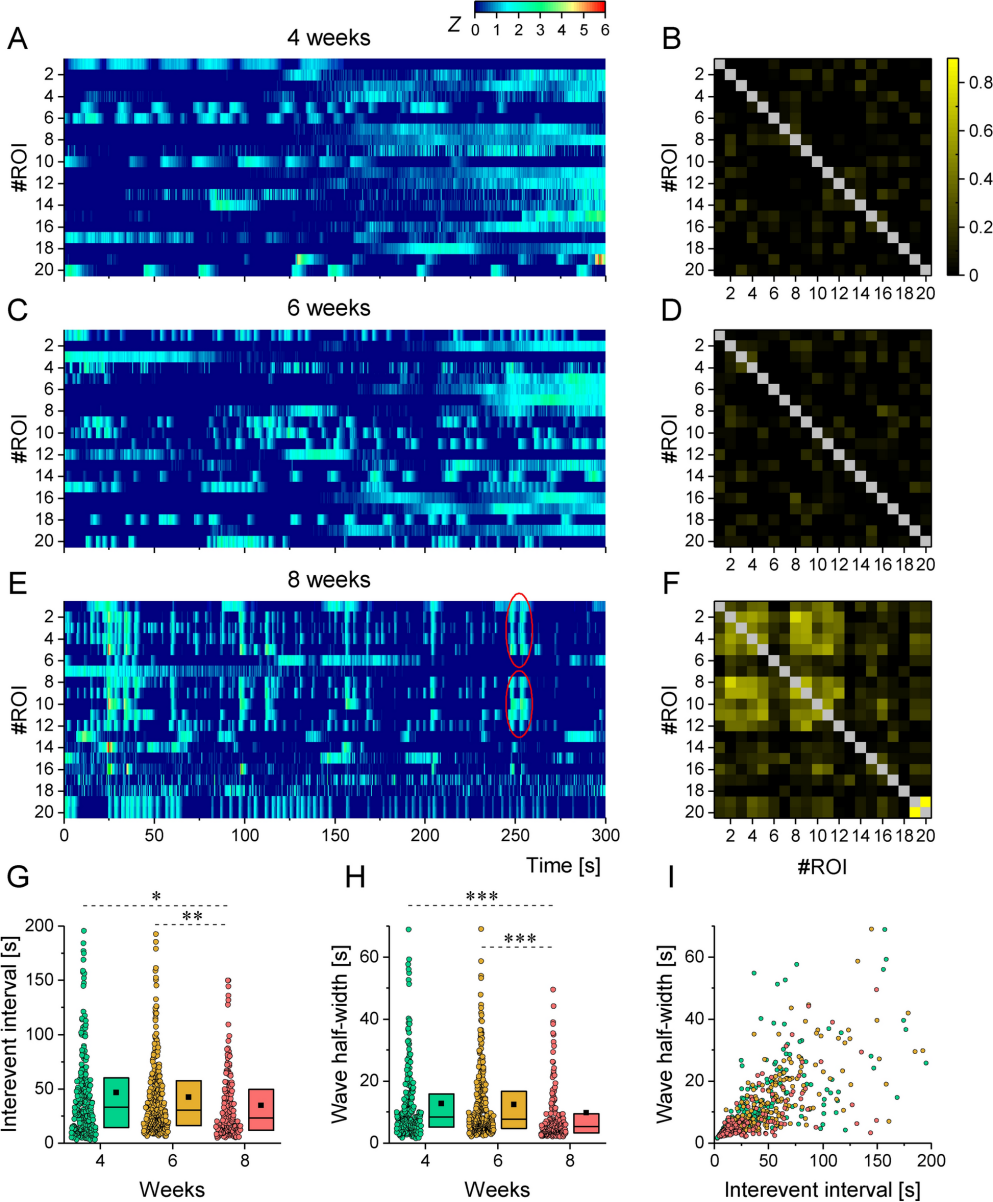
Spontaneous Ca^2+^ transients become more prominent in h-iPSC-Ns during maturation. (**A**), (**C**) and (**E**) Representative heatmaps of the Fluo-3-AM spontaneous Ca^2+^ transients at 4, 6 and 8 weeks of h-iPSC-Ns maturation, respectively. Data from a single microscopic view consisting of 20 Regions of Interest (ROIs) are visualized using the ΔF/F0 ratio, which represents the change in fluorescent intensity (ΔF) normalized to the baseline fluorescence of Fluo-3 (F0). Synchronized Ca^2+^ signals in a few regions of interest (ROIs) are encircled in (**E**). Results are depicted using a standard z score (0–6) and a color scale, where 0 denotes the lack of a Ca^2+^ transient and 6 represents the maximum of the provided collection of calcium transients. (**B**), (**D**) and (**F**) Cross-correlations between the activities of the same set of 20 regions of interest (ROIs) are displayed in a symmetric matrix. Colors vary based on the Spearman correlation, where a value of 0 indicates non-correlation and 1 indicates high correlation. (**G**) The interevent interval of calcium transients was determined at 4, 6, and 8 weeks of maturation. The median interevent interval of calcium transients gradually decreased during maturation. Calcium transient halfwidth (wave halfwidth) exhibited similar behavior (**H**). We established a Spearman correlation between the calcium transient halfwidth and interevent interval (**I**), revealing a time-independent correlation of 0.78 where the data points were labelled in a similar manner to those on G and H. Data were acquired from 3 independent cultures and are presented as box plots of interquartile range together with individual data points (n = 232, 280, 147), respectively at week 4, 6 and 8. Pairwise comparisons were executed using one-way non-parametric ANOVA with *p* < 0.05 (*), *p* < 0.01 (**) or *p* < 0.001 (***).

To evaluate the neuronal circuit forming capacities of h-iPSC-Ns over time, spontaneous calcium signals were recorded during neuronal maturation. Neurons were loaded with Fluo-3-AM dye and neuronal activity was recorded at 4, 6 and 8 weeks. Relative changes of intracellular Ca^2+^ level within the soma of individual neurons were plotted as heatmaps (Fig. 6A,C,E) or normalized as correlation matrices (Fig. 6B,D,F). Calcium transients within 4- or 6-week-old cultures were non-synchronized, revealing a randomized neuronal activity (Fig. 6A–D). On the other hand, neuronal activity was partially synchronized in 8-week-old cultures (Fig. 6E,F). The frequency of calcium waves remained relatively stable over 4 to 6 weeks, but a significant decrease in the interevent intervals (Fig. 6G) indicated the appearance of recurring Ca^2+^ wave multiples (bursts) in 8-week-old cultures. At this age, the decrease of the event half-width parameter was observed (Fig. 6H) that is consistent with the development of faster decaying Ca^2+^ signals. Indeed, nearly half of the ROI channels displayed recurring Ca^2+^ waves that indicated the formation of synaptically interconnected neuronal circuits. When a Spearman correlation was conducted between the halfwidth of the calcium transient and the interevent interval, it yielded a time-independent correlation coefficient of 0.78, indicating that the two parameters vary in parallel over time (Fig. 6I). Thus, our correlation analysis revealed the development of network synchronization in cultures only by 8 weeks of maturation.

## Discussion

The application of neurons derived from human induced pluripotent stem cells (h-iPSC-Ns) as a reliable model for neuronal differentiation and drug development has been confirmed by numerous studies^2,5,29^. Since the introduction of the Yamanaka protocol^30^, various combinations of reprogramming, differentiation and maturation protocols have been developed to direct the reprogrammed cells towards a reliable and robust neuronal phenotype. Consequently, there is a need to facilitate and track neuronal differentiation via standardized methods to characterize the maturation variability/heterogeneity of induced neurons, as reviewed by^31^. This can be accomplished by establishing a framework of parameters to identify neuronal developmental traits, with specific emphasis on cell-autonomous and network attributes. Such guidelines can provide a valuable tool to establish targeted experiments (e.g. drug discovery) that are adapted to specific stages of neural maturation.

In the present study, we monitored the maturation of h-iPSC-Ns induced by the 2-inhibitor method for up to 10 weeks by combining multiple analyses such as immunocytochemistry, morphometric essay, patch clamp using conventional current step protocols, dynamic clamp, calcium imaging, as well as monitoring expression changes of GABA_A_ receptor subunits and chloride transporters by qRT-PCR. Some changes—including the expression of specific neuronal markers, passive membrane or network properties—suggest a linear progression of maturation, whereas other parameters, such as morphometric maturation or the appearance of active membrane properties, showed biphasic evolution. Interestingly, excitability responsiveness did not change during development. In addition, the biphasic properties partially overlapped with each other in time. This phasic state of maturation could be explained by poised neuronal maturation genes (also called “effectors”), such as synaptic proteins and ion channels, as well as other chromatin regulators, which “competitively” interact with epigenetic barriers^32^.

In our timeframe, the development of electrophysiological properties occurred while neuronal arborization was still relatively simple. There was a linear progression of increasing conductance and decreasing membrane resistance of the developing human neurons. On the other hand, active membrane parameters such as the half-width and amplitude of action potentials showed a step-like change of the excitability properties with an inflection point at week 5. We also noticed that the maturation of h-iPSC-Ns in our experiments remained very consistent in respect to their overall cellular properties. While the variability of membrane resistance and spike shape parameters were significant, especially during early measurements, regular firing neurons exhibited typical features, which remained stable during week 6 to 10. Interestingly, the presence of the *kink* in the phase portraits of action potentials preceded the stabilization of active parameters but was prominent and appeared in parallel with the firing phenotype from week 5. We introduced this parameter in order to characterize the degree of temporal mismatch between the activation of somatic and axon initial segment (AIS) related Na^+^-currents. Pronounced kink in the phase portrait of action potentials reliably indicates the activation of low-threshold Na^+^-currents in the AIS followed by the rise of slightly elevated threshold Na^+^-currents in the perisomatic region. Due to the variability in the shape and magnitude of kink in the phase portraits it is somewhat challenging to establish a parameter that accurately describes this phenomenon as a single scalar measure. Yet, we found the above defined kink latency as an informative parameter, and we interpret its stabilization after week 5 as the sign of stabilization of the AIS relative to the soma. Follow-up immunocytochemical investigations can verify this notion. We also acknowledge that detection and quantification of kink in low amplitude action potentials (spikelets), characteristic of maturing neurons during the 1st and 2nd week, are rather limited. Hence, kink latency values are largely missing in the 1–4-week-old measured cells.

It is noteworthy that more intricate physiological features such as inward rectification, post-inhibitory rebound and burstiness were not observed in our patch clamp recordings. This indicates the lack or low level of certain voltage-dependent currents such as the hyperpolarization-activated cation current (I_h_) or the low threshold Ca^2+^-current (I_T_)^33^. Our dynamic clamp experiments also revealed consistent firing responses and clear differences in spike timing reliability under low vs. high frequency inputs. This was not surprising, as the membrane time constant of such developing neurons remains high (approximately 100 ms) while the maximum spiking frequency is well under 20 Hz. However, neurons can still participate in network oscillations at lower frequencies (4 Hz or less), and this can contribute to the development of synchronized network activity such as bursting.

According to data published by Odawara et al., long-term cultivation of h-iPSC-derived cortical neurons cultured on an astrocyte feeder layer resulted in a progressive increase in average firing frequency between week 2 and 34. This indicates a doubling in spike frequency between week 5 and 6^34^, with a switch to a more mature phenotype resembling fetal brain tissues after 6 weeks^35^. Comparable results were observed regarding maturation timepoints in our h-iPSC-Ns, where a significant increase in neuronal maturation between 4 and 6 weeks was evident based on both passive and active electrophysiological properties. Our data and previously published studies indicate that a minimum of 4–5 weeks of maturation is needed to attain a fully stabilized electrophysiological phenotype^34,35^. Comparable outcomes for the active and passive membrane properties of the human neurons were also reported in other studies. For example, a decline in membrane resistance and membrane time constant were reported^36,37^, which is consistent with the timeline of neuronal development over 4–8 weeks. Notably, the expression of voltage-gated sodium channels increased during neuronal maturation, and the membrane became capable of producing action potentials (AP) following depolarization, as observed by Kawaguchi et al.^38^. In addition, the halfwidth of the APs decreased and the amplitude of the AP increased over time, as also indicated by our data.

It was also interesting that changes in active membrane properties preceded the maturation of cell morphology in time. The initial neurite extension was slowly followed by further branching, so dendritic arborization accelerated only after the 5th week of cultivation, resulting in a more complex cell morphology from the 6th week on. Detailed analyses of morphological maturation of iPSC-Ns also indicated that the neurites of cultured neurons increase in length from DIV 3–9, but both the area of the soma and the length of neurites decreased on subsequent development days^39^. Our morphological analysis revealed a biphasic change in the total dendritic length and process extension between the first 5 and later weeks of maturation, preceding the time-correlated network activity at week 8. The occurrence of the calcium transients highlighting the synchronicity of the neuronal network also increased in a time dependent manner between the 4 to 8 weeks, which is consistent with the development of the electrophysiology parameters^36^.

Network activity was based on the development and action of abundant fast glutamatergic and GABAergic synaptic connections, both imposing depolarizing action on neurons, similarly to in vivo neural development^40^. Although a significant change in the expression of GABA_A_ subunits was observed during the 8-week-long maturation of the cultures, the shift from depolarizing to hyperpolarizing GABA-action did not yet occur. Comparable results of GABA_A_ receptor subunit expression were observed in iCell iPSC-Ns, based on a combination of *α*1/2, *β*1/3, and *γ*3 subunits^41^. Yuan et al. revealed that iCell-Neurons contain a moderate amount of *γ*2 mRNA which—together with *α*5 and β3 subunits—form a unique neuronal subtype distribution when compared to the adult human brain^42^. This is due to the fact that *α*5 subunits are present in less than 5% of all GABA_A_ receptors in the brain, while *α*1 subunit is present in nearly 60%^43^. In contrast, our developing neurons gradually expressed the necessary subunits for the GABA_A_ receptors as *α*2, β3, and γ2/3 subunits were dominant together with the time-increasing manner of *α*1. Interestingly, shifting the expression from early expressed *NKCC1* to *KCC2* transporters did not occur during the 8-week-long cultivation.

Taken together, our study provides a thematic scheme about the maturation of human induced pluripotent stem cell derived neurons via combining all the prominent key markers generally used to characterize electrophysiological, morphological or molecular development. We provide evidence that in order to establish the most appropriate read-out time of the planned experiments (useful e.g. in testing drug pharmacology), the right level of neuronal maturation should be determined with a comprehensive approach. Importantly, electrophysiological responsiveness does not guarantee solely the appearance of all other mature integrative properties. In addition, seemingly more complex morphological phenotypes do not infer the presence of robust network connectivity. Therefore, a given set of these observations should be used as follow-up standards of the h-iPSC-Ns maturation providing a powerful tool to reliably characterize given stages of neuronal maturation.

## Supplementary Information

Supplementary Information.

## Data Availability

All data presented in this study are available from the corresponding author upon reasonable request.
