# Supplementary material for: Multifactorial approach is needed to unravel the maturation phases of human neurons derived from induced pluripotent stem cells
Source: Sci Rep. 2025 Jan 21;15:2627. doi: 10.1038/s41598-024-81140-4 (PMC11751176; doi:10.1038/s41598-024-81140-4)
Supplement: Supplementary file 1 — Supplementary Information. [file 41598_2024_81140_MOESM1_ESM.pdf]

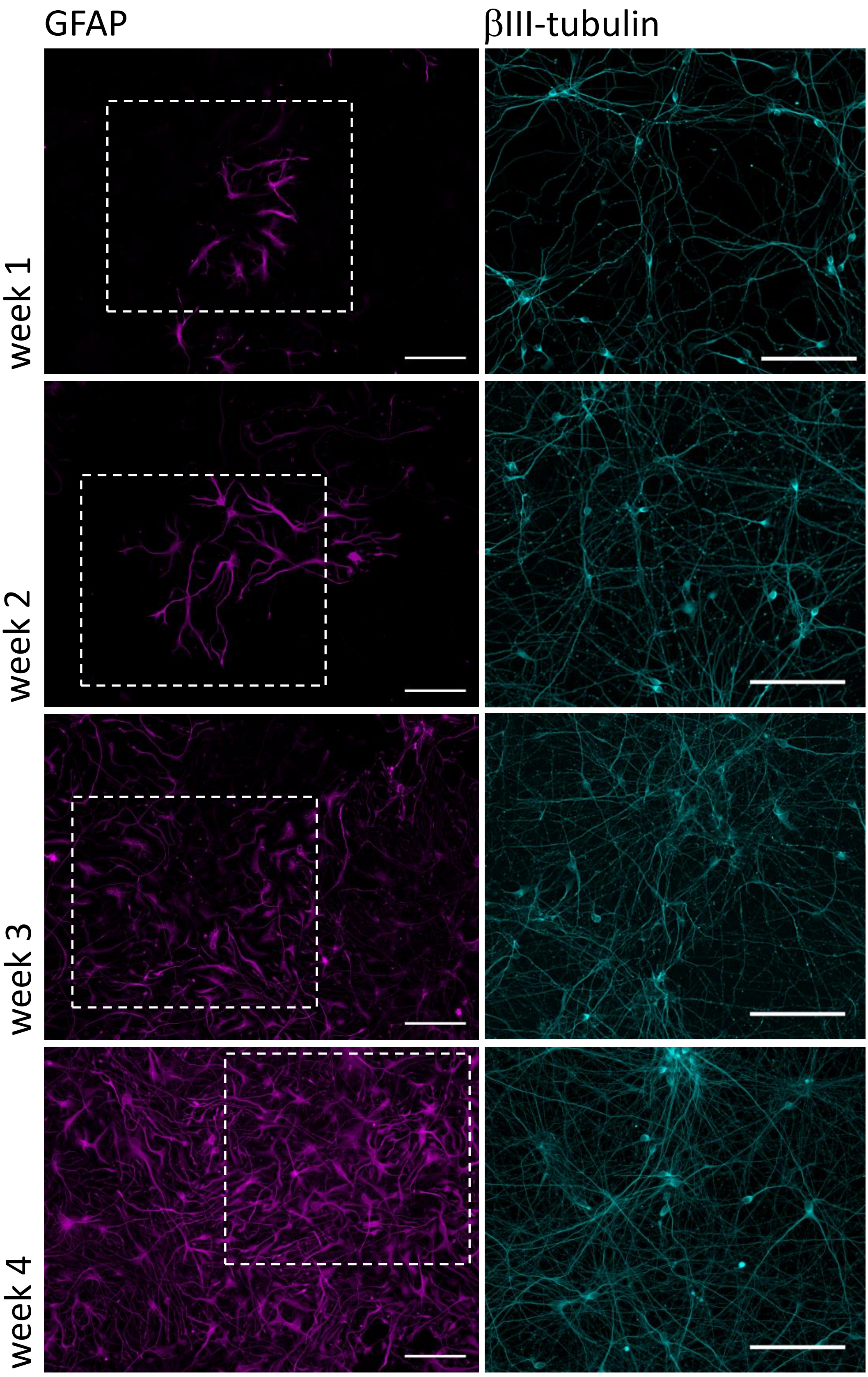

Supplementary figure 1. Progressive differentiation of neurons vs. glia population in a time dependent manner. Both βIII-tubulin and GFAP markers show a progressive differentiation of neurons and glia cells from the first week to the 4th week of differentiation. The dashed areas within GFAP stained images (left) are enlarged on the right, presenting βIII-tubulin positive neuronal networks. Scale bar: 100μm.
